# Efficacy of antiviral drug AV2 in the treatment of human papillomavirus-associated precancerous lesions of the uterine cervix: A randomized placebo-controlled clinical trial in Kinshasa, DR Congo. (KINVAV study)

**DOI:** 10.1016/j.conctc.2017.09.008

**Published:** 2017-09-28

**Authors:** Alex Baleka Mutombo, Rahma Tozin, Cindy Simoens, Ramokone Lisbeth, Johannes Bogers, Jean-Pierre Van Geertruyden, Yves Jacquemyn

**Affiliations:** a Department of Obstetrics and Gynecology, Kinshasa University Hospital, Kinshasa, Democratic Republic of the Congo; b Applied Molecular Biology Research Group (AMBIOR), Faculty of Medicine and Health Sciences, University of Antwerp, Universiteitsplein 1, 2610, Wilrijk, Belgium; c HIV and Hepatitis Research Unit, Department of Virology, Sefako Makgatho Health Science University, Pretoria, South Africa; d Global Health Institute, Faculty of Medicine & Health Sciences, University of Antwerp, Doornstraat 331, BE-2610, Wilrijk, Belgium; e Department of Obstetrics and Gynaecology, UZA Antwerp University Hospital, Wilrijkstraat 10, 2650, Edegem, Belgium

**Keywords:** Human papillomavirus, Precancerous lesions, Uterine cervix, Antiviral, Randomized clinical trial, Democratic Republic of the Congo

## Abstract

**Background:**

Cervical Cancer (CC) is a major public health problem in DR Congo; the high incidence of CC is due to the inexistence of effective screening programs based on cytology and/or HPV detection followed by appropriate treatments. This situation highlights the need to implement efficacious and inexpensive treatment methods. This study aims at evaluating the efficacy of a topical antiviral drug named AV2^®^ as a treatment for HPV-associated lesions of the cervix.

**Methods:**

Women will undergo cytology sampling, HPV testing and Visual inspection of the cervix after application of 5% acetic acid (VIA). VIA-positive women will be randomized to one of two groups to receive treatment by either AV2^®^or placebo. They will undergo control examinations after two months and after six months. In case of persistent lesions on VIA, treatment by cryotherapy will be done. The primary outcomes will be the change of lesions, the clearance of HPV DNA, and the correlation of the two 2 months after treatment with AV2^®^.

**Conclusion:**

This study is the first large-scale study in Africa to evaluate systematically the efficacy and safety of a topical antiviral drug for the treatment of HPV— associated lesions of the cervix. Its findings will direct the planning of suitable algorithms for CC screening and treatment.

**Clinical trial registration:**

ClinicalTrials.gov – Unique identifier: NCT02346227, registered on November 8, 2014.

## 1 Introduction

Cervical cancer (CC) is the third most common cancer in women worlwide with an estimated 530 000 new cases per year. It is the most common cancer in women of Low and Middle Income Countries (LMICs) [1].

In the Democratic Republic of the Congo (DRC), 18.85 million women, aged 15 years and older are at risk of developing CC. Current estimates indicate that every year 6024 women are diagnosed with cervical cancer and 4719 die from the disease [2].

The link between human papillomavirus (HPV) infection and CC is well established [3]. HPV infection is implicated in 99.7% of CC cases worldwide [4], [5]. When HPV infection persists in the cervix, it can induce changes which constitute the precancerous lesions of the cervix (PLC), also called dysplasia or cervical intraepithelial neoplasia (CIN)·CIN is asymptomatic and progresses to CC over a prolonged period of time (7–20 years). CC can be prevented by screening and treatment of CIN in asymptomatic women. Cytology is considered as the standard CC screening method but is not feasible in LMICs due to its high cost, lack of adequate infrastructure or qualified cytotechnicians. Therefore, visual inspection of the cervix after application of 5% acetic acid (VIA) is recommended as a screening tool in low-resource settings with no acces to HPV testing or cytology screening. It has limited specificity but a high sensitivity, is cost-effective and provides immediate results [6], [7].

Sauvaget et al. performed meta-analyses with different categories of studies to provide an updated estimation of the accuracy of visual inspection with acetic acid (VIA) in detecting true disease. The reference category consisted of 26 studies in which VIA was performed on asymptomatic women who all underwent confirmatory testing and in which the disease threshold was cervical intraepithelial neoplasia grade 2. They reported an 80% sensitivity (range, 79%–82%) and a 92% specificity (range, 91%–92%) for VIA. Study region, capacity of screener, or size of the study population did not modify VIA accuracy. In conclusion, it was shown that screening for precancerous and cancerous cervical lesions using VIA is a simple, low-cost, and efficient alternative to cytologic testing in low-resource areas. The more the severity of disease, the more sensitive and specific is VIA in detecting the disease [8].

In another meta-analysis by Sauvaget, Cryotherapy was shown to be an effective, safe, and acceptable treatment for CIN. Actually, on a equivalent of 28,827 cases of treated CIN, Cryotherapy achieved cure rates of 94.0% for CIN1, 92.0% for CIN2, and 85.0% for CIN3 [9].

Of all available and effective treatments of CIN, cryotherapy seems the most appropriate for LMICs, but is still hampered by major logistical problems such as unavailability of refrigerant gas. These feasibility issues highlight the need for a low-cost and minimally invasive method of treating CIN in LMICs [7].

Recently, an antiviral drug called Antiviral 2 (AV2^®^, Cesa Alliance, Luxembourg) made of natural essential oils was developed and preliminary results suggest it to be effective in clearing or reducing the size of PLC related to HPV [10]. The Food and Drug Administration (FDA) approved its organic compounds (carvone, eugenol, geraniol, and nerolidol). These combined compounds have a broad spectrum anti-viral effect, both topically and orally [11] and AV2 may regress cervical lesions due to deactivation of the virus.

In a recently published phase 2 clinical trial study conducted by Martinez et al. at Hospital de la Familia, in Juarez, Mexico, efficacy of AV2^®^ in the regression of cervical lesions diagnosed at colposcopy was compared to placebo in 50 women over the age of 18. The results showed that the application of AV2^®^ yielded a reduction of more than 50% of lesion size for 21 out of the 28 (75%) patients who received the active treatment, versus 0% for the comparable placebo pool. At the other end of the scale, only 2 (7%) participants in the AV2^®^ group failed to respond positively compared to 80% of participants in the placebo group [12]. Medical treatment of precancerous lesions of the cervix with AV2^®^ could offer minimally invasive treatment with minor morbidity and time constraints.

## 2 Methods/design

### 2.1 Study design

The present study is a double-blind randomized placebo-controlled clinical trial. It will include 400 patients in a health centre in Kinshasa, DRC: Centre Hospitalier du Mont-Amba. It is estimated to last 56 weeks considering final data collection for primary outcome measurements.

### 2.2 Patients

Inclusion criteria: The targeted population is all sexually-active women, aged at least 25 years old and who will give their informed consent to participate in the study.

The reason for this target population is based on the natural history of HPV infection. HPV is acquired at the onset of sexual activity which occurs at around 15 years of age. Its prevalence is very high at a younger age and is often transient because most women will spontaneously eliminate the virus after some years. When it persists, HPV infection can take in average 10 years to lead to CC. Thus, testing is projected to start 10 years later after sexual debut in this case at 25 years old onward.

Exclusion Criteria: Pregnant, breastfeeding and post-partum women will not be accepted in the study. In case of suspected or diagnosed cervical cancer, the women will be referred in a tertiary hospital for the proper management. Other exclusion criteria include: previous cervical surgery, medical history of any severe diseases, intake or application of other antivirals, known or suspected allergic or adverse response to the investigational product or its components and women who are unable to follow the study protocol.

### 2.3 Procedures and follow-up (Fig. 1)

Prior to the start of the study, a basic training of the doctors and nurses involved will be organized. This training will encompass Good Clinical Practice (GCP), collection of liquid-based cytology sample with cytobrushes, and VIA and cryotherapy procedures.

Women aged 25 and over will be invited to participate in a community cervical cancer screening programme. After giving their informed consent, eligible women will be examined by a trained doctor. The procedure will be performed while the woman lies in the lithotomy position on the examination table. After the cervix is exposed by a speculum inserted in the vagina, the doctor will collect a LBC specimen using the Thinprep PreservCyt solution^®^ (Hologic, Marlborough,USA), immediately after LBC sampling, VIA will be performed. Its results will be reported according to the WHO criteria and will serve for randomization. The results for LBC and HPV testing will not be available at this moment.

VIA-negative women will not be randomized and will receive no treatment. VIA-positive women will be randomized to one of two groups to receive either AV2^®^ or placebo. All randomized women will be followed-up and recalled after two months for repeat LBC and VIA. Women remaining positive on VIA at this stage will be treated by cryotherapy.

At six months' follow-up, women will be assessed in the same manner regarding VIA and LBC. The study will end at this stage. At each stage, the examinators will be blind to the previous results concerning the patient. At the end of the study, All women who will test positive for cervical lesions or HPV will be properly treated according to standard protocols and resources.

### 2.4 Randomization and blinding

**Fig. 1:**
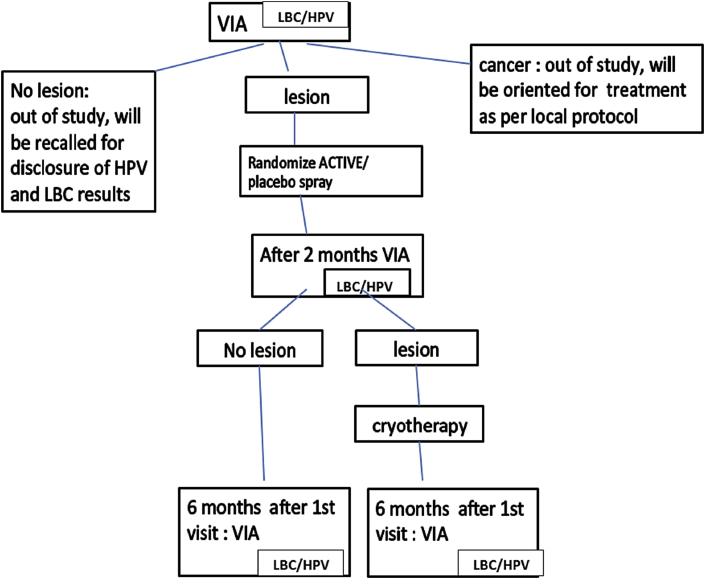
Study design.

VIA positive women will be randomized in a block wise manner, without stratification, to one of two treatment arms as shown in Fig. 1. Patients and study investigators will have no knowledge of the treatment arm allocation. To maintain blinding, active drug and placebo glass containers will be identical in appearance and scent. Patients and investigators will be unblinded only in the event of a suspected serious adverse reaction.

### 2.5 Visual inspection of the cervix with acetic acid

VIA involves the painting of the cervix with 5% acetic acid and the observation of the color change 1 min after. The most common form of reporting the VIA test outcome involves negative and positive categories, depending upon the absence or presence of acetowhite lesions and clinical signs of invasive cancer. A positive test is generally based on the detection of well-defined, densely opaque acetowhite lesions in the transformation zone closer to the squamocolumnar junction. The criteria for a negative test includes one or more of the following: no acetowhite lesions, faint ill-defined translucent acetowhite lesions, endocervical polyps, nabothian cysts, dot-like acetowhite lesions, acetowhite lesions far away from the transformation zone and a prominent squamo-columnar junction.

### 2.6 Methods for LBC samples

It is not possible to perform HPV testing locally as there are no laboratory facilities in DRC allowing this. Laboratory examinations will be done at the HPV and STIs Training Centre for Africa, Sefako Makgatho Health Sciences University, Pretoria, South Africa. To ensure quality, all the samples will be re-examined at Algemeen Medisch Laboratorium (AML) in Antwerp, Belgium.

From a single LBC specimen collected in the ThinPrep test vial containing the PreservCyt^®^ solution, both cytology and HPV testing will be performed. HPV testing in this study will encompass both HPV detection and HPV genotyping as well as HPV viral particle load. Cytology results will be reported according to the 2001 Bethesda system terminology.

Sample collection

The HPV and STIs Training Centre for Africa will receive Thinprep vials with 10 ml of the cervical sample. The samples will be stored at room temperature between 15 °C and 30 °C in a store room. The level 2 safety hood is set up and prepared by cleaning with alcohol followed by DNASe away for decontamination. The vials will be moved to dirty area (extraction room) for preparation. The pipettes will also be cleaned with alcohol and DNASe away for decontamination. The 2 ml tubes will be labeled according to the sample number on the ThinPrep vials. A 1000 μl pipette will be used to remove 2 ml of sample from the ThinPrep vials into the 2 ml test tubes for HPV testing avoiding any spillage or aerosol. The ThinPrep vials will be then filled to the ThinPrep vial mark (10 ml) with Preservcyt solution to prepare for cytology slide processing. The ThinPrep vials will then be capped and stored at room temperature until processing of the cytology slides on ThinPrep T5000 system.

#### 2.6.1 Liquid-based cytology

Preparation of the cytology slides will be performed from the cervical samples in ThinPrep Preservcyt^®^ solutions using the ThinPrep T5000 cytology system. Labeled slides with corresponding number on the Vial together with a filter and the vial are placed on the carousel for processing. Once prepared, the slides will be stained and covered with cover using entelen. After staining, the slides will be screened by a qualified and trained cytotechnologist and reviewed by Pathologist. All the results will be reported on a form using the vial number only as identifier.

#### 2.6.2 HPV testing

We will use the Abbott system. The Abbott RealTime High Risk HPV is a qualitative, multiplex real time PCR for the detection of DNA from 14 High Risk HPV genotypes 16, 18, 31, 33, 35, 39, 45, 51, 52, 56, 58, 59, 66, and 68 in clinical specimens, typing of HPV 16 and/or HPV 18, single and mixed infections (Abbott Molecular, Wiesbaden). An endogenous human beta globin sequence is detected as sample validity control for cell adequacy, sample extraction and amplification efficiency. Two external controls (negative and positive controls) are included in every run. The viral load will be expressed as the number of HPV copies per million of normal human cells.

### 2.7 Investigational product

AV2^®^ is a synergistic combination of FDA-approved organic compounds, having a highly effective broad-spectrum anti-viral effect, both topically as orally. It is a mixture of natural essential oil components (carvone, eugenol, geraniol, nerolidol) in equal volumes diluted 50% in olive oil (Olea europea).

The components of AV2 have a long history of use in the Food and Fragrance industry. The components earned the GRAS (Generally Recognized As Safe) status in 1965. The present FDA status of the components is the following: Carvone: GRAS under 21 CFR 182.60; Eugenol: “Direct Food Substances Affirmed as Generally Recognized as Safe” status under 21 CRF 184.1257; Geraniol: GRAS under 21 CFR 182.60; Nerolidol: “Food Additives Permitted for Direct Addition to Food for Human Consumption”” status by FDA under 21 CFR 172.515 [13], [14], [15].

It is postulated that AV2^®^ de-activates the HPV virus outside the cell by preventing endocytosis. Due to the broad-spectrum antiviral activity of AV2^®^, cervical lesions may regress due to deactivation of the virus. (8). It is administered as a topical spray to the cervix.

*Side effects:* Topically AV2^®^ does provoke for about 30–60 s a warming or burning feeling similar to disinfecting alcohol on very sensitive areas such as *Labia minora*.

*The Placebo* consists of 10% lemon (*citrus limon*) and 10% lime (*citrus aurantifolia*) essential oils in 80% olive oil. The oils are included to provide a fragrance to the placebo, similar to AV2^®^.

### 2.8 Outcomes

- The primary outcome will be the change of lesions 2 months after randomization.

Change of lesions will be defined as regression or progression of the size of the lesion seen on VIA. Regression can be partial or complete when there is a total disappearance of the lesion also called remission. On cytology, regression will be defined as improvement from high-grade to low-grade CIN and remission will be defined as improvement from any grade of CIN to no CIN.

- The secondary outcomes will be:The secondary outcomes will be
  • Clearance of HPV at 2 months post-randomization,
  • Correlation between change of lesions change in HPV DNA at 2 months post-randomization,
  • Change in HPV viral particle load 6 months post-randomization.
- The tertiary outcome will be the number of patients with adverse effect.

An adverse effect (AE) will be defined as any unfavourable and unintended sign, symptom, or disease temporally associated with the use of the product administered. A causality assessment of the AEs will be done in accordance with the guidelines of the WHO–Uppsala Monitoring Centre.

### 2.9 Sample size

Assuming a lesion regression rate of 50% in the non-interventional group and 70% in the antiviral drug group at 2 months post-treatment, a minimum sample size of 163 patients is needed to show with 95% power a difference between both groups. To allow for up to 10% drop-outs or unevaluable patients, we will recruit at least 190 patients in each arm. We expect 20% of the women to be HPV positive, of whom 10% with and 10% without lesions, indicating that over 1900 women will have to be enrolled to obtain the required sample size.

### 2.10 Statistical analyses

Data will be double-entered and cleaned in EpiInfo (version 6.04 b, Centers for Diseases Control and Prevention). Proportions will be compared using the x^2^ or Fisher's exact tests (when required). Student's *t*-test will be used for continuous variables. A Mc Nemar or paired *t*test will be used for within-patient comparisons where approriate. Non-normally distributed variables will be transformed, or nonparametric tests (Wilcoxon or Kruskal-Wallis) will be used. Stepwise multivariate analyses will be performed, and all possible interactions up to order 2 will be tested. All reported *P* values will be 2-sided. For all statistical analyses a *P* value of ≤0.05 will be considered as statistically significant.

All analyses will be performed using the STATA statistical analysis software packages (Version 12; Stata Corp, Lakeway, College Station, Texas, USA).

### 2.11 Ethical issues

The KINVAV study has been approved by the Institutional Ethical Committees /Institutional Review Board (IEC/IRB) of both the University of Kinshasa School of Public Health (Kinshasa, DRC) and the Antwerp University Hospital (Antwerp, Belgium), and registered in ClinicalTrials.gov (NCT02346227). It will be conducted in full agreement with the principles of the “Declaration of Helsinki” and subsequent relevant amendments. All participants will provide written informed consent before entering the trial.

## 3 Discussion

In this trial, we will randomize women based on VIA which is known to be a subjective test. But according to the WHO studies, VIA does show better performance that allow its use as a cervical cancer screening test, especially in the frame of « see and treat approach » (SAT) [16], [17], [18].

For our study, the ideal process should be to start with HPV testing and secondarily randomize only HPV positive women. Unfortunately, real time HPV test such as Care HPV^®^ test (Qiagen) is not available yet in DRC. Plus, we need to prioritize the SAT approach which minimizes lost to follow-ups and all the difficulties encountered when women have to be recalled for results. For instance, in Africa, mothers are the pivot in the life of the family, they go to the crops, they travel for small businesses and all these situations possibly tend to affect follow-up rates.

Moreover, in our institution we are not able to perform HPV testing needed in this study yet. The samples will be shipped to South Africa for both LBC and HPV testing and it will take some months to get the results back. So, we cannot rely on HPV testing for randomization. We will thus randomize based on VIA test whose results are immediate. According to the natural history of cervical cancer, it takes 7–20 years for cervical HPV infection or dysplasia to lead to cervical cancer and a high proportion of HPV infection and mild dysplasia may regress spontaneously. During the two-months follow-up period we will detect all women with cervical dysplasia, even in the placebo arm, and treat them accordingly by cryotherapy which is a standardized treatment method used for the treatment of precancerous lesions. For those reasons, placebo can be safely used.

This study will be the first large-scaled study in Kinshasa to report on HPV prevalence and genotyping in DRC. One limitation for this study is the fact that instead of cytology or hpv testing results, randomization will be based on VIA which is a subjective test.

Finally, when we will get all the results concerning LBC and HPV testing, we will recall women to inform them of their results and treat those in need with standard treatment until we clear all precancerous lesions.

## Publication plan

Sources of funding, institutional affiliations and conflicts of interest will be declared in the subsequent publications related to this study.

## Competing interests

The authors declare that they have no competing interests.

## Author's contributions

ABM, YJ, JPVG have written and designed the study protocol. JB, CS, RT, RL have commented and delivered expert opinion on the protocol. ABM IS site principal investigator. YJ is coordinating the trial on behalf of the sponsor. All authors read and approved the final manuscript.

## Trial status

Recruiting

## Abbreviations list

AE: Adverse Event
AV2: Antiviral 2
CC: Cervical Cancer
CIN: Cervical Intraepithelial Neoplasia
CRF: Case Report Form
DNA: Deoxyribonucleic acid
DRC: Democratic Republic of the Congo
FDA: Food and Drug Administration
GCP: Good Clinical Practice
HIC: High-income countries
HPV: Human Papillomavirus
ICH: International Conference on Harmonization
IEC: Institutional Ethics Committee
IRB: Institutional Review Board
LBC: Liquid-based cytology
LMICs: Low-income countries
PLC: Precancerous Lesions of the Cervix
PI: Principal Investigator
PCR: Polymerase Chain Reaction
SAE: Serious Adverse Event
SAT: See-and-Treat
SOPs: Standard of Procedures
VIA: Visual inspection with 5% Acetic Acid
WHO: World Health Organization
